# Limited ant co-occurrence and defensive mutualism in *Acacia* plants in a West African savanna

**DOI:** 10.1093/aobpla/plab036

**Published:** 2021-06-03

**Authors:** Anyse Djogbenou, Akomian F Azihou, Anicet G Dassou, Achille E Assogbadjo, Barthelemy Kassa, Orou G Gaoue

**Affiliations:** 1 Laboratory of Applied Ecology, Faculty of Agronomic Sciences, University of Abomey-Calavi, 01 BP 526, Cotonou, Benin; 2 Laboratory of Biotechnology, Genetic Resources, Plant and Animal Breeding, Faculty of Sciences and Technology of Dassa, National University of Sciences, Technology, Engineering and Mathematics, 01 BP 14, Dassa-Zoumè, Benin; 3 Department of Ecology and Evolutionary Biology, University of Tennessee, Knoxville, TN 37996, USA; 4 Faculty of Agronomy, University of Parakou, BP 123, Parakou, Benin; 5 Department of Geography, Environmental Management and Energy Sciences, University of Johannesburg, APK Campus, Johannesburg, South Africa

**Keywords:** *Acacia* species, ants, Dahomey Gap, defensive mutualism, elephant damage, plant–animal interactions, West African savanna

## Abstract

Our understanding of the role of fire and effect of ant species composition, beyond their diversity and abundance, on the effectiveness of mutualism defence is limited. Most of our knowledge of ant–plant defence in tropical Africa is biased towards East African savannas which have richer soil, higher primary productivity and a more diverse arthropods and mammal community than West African savannas. We assessed the diversity of ant species associated with *Acacia* species in the Pendjari Biosphere Reserve in the Dahomey Gap, and their impacts on elephant damage. Elephant damage, ant diversity and abundance were measured in stands of five *Acacia* species. Eleven ant species were identified in the *Acacia* stands. The composition of these ant communities varied across *Acacia* species. Pair of ant species co-occurred in only 2 % of sampled trees, suggesting a strong competitive exclusion. Within this annually burnt environment, ants were rare on small trees. The intensity of elephant-caused branch breaking did not vary between trees with ants and trees without ants, suggesting limited *Acacia*–ant mutualism. Such limited biotic defence may mask strong physical and chemical defence mechanisms of *Acacia* trees against elephant damage. Ant assemblages in West Africa, unlike those in the more productive East Africa, are particularly species-poor. However, there is a convergence between these two regions in low rate of ant co-occurrence which might indicate strong competitive exclusion. Our study suggests that such low ant species richness while limiting the efficacy of mutualism in controlling mega-herbivore damage may mask a strong defence syndrome.

## Teaser

We know particularly little about species interactions in most African ecosystems, and this clearly limits the ability of ecologists to conduct global analyses without criticism of a tropical bias. Here, we provide one of the rare studies on plant–ant interactions in West Africa, studying *Acacia*–ant interactions in the Pendjari Biopshere Reserve. We found only 11 ant species dwelling on five *Acacia* species that we studied. These ant species tend to avoid being present on the same species or tree. Such species-poor ant assemblages provide weak defence against elephant damage on the *Acacia* trees.

## Introduction

In African savannas, elephants (*Loxodonta africana*) confined to protected areas are a major driver of savanna dynamics, impacting trees through browsing, bark stripping and uprooting (hereafter called ‘elephant damage’) (Asner and Levick 2012; Kassa *et al.* 2014; Morrison *et al.* 2016; Salako *et al.* 2016). Elephants disproportionately damaged certain tree species relative to their availability on the landscape as it is the case for *Adansonia digitata*, *Borassus aethiopum* (Salako *et al.* 2016) and *Acacia senegal* (Morrison *et al.* 2016). Many strongly selected species engage in defensive mutualism with ants that are effective in reducing browsing by elephants (Martins 2010; Stanton and Palmer 2011; Palmer and Brody 2013). Some *Acacia* trees provide both nest space (‘swollen thorn’ domatia) and carbohydrate-rich extra-floral nectar for ants, and ants provide protection from vertebrate herbivore (Palmer and Brody 2007). In Africa, most evidence of this important mutualism comes from *Acacia drepanolobium*-dominated savannas in protected areas of East Africa (Palmer and Brody 2013; Kimuyu *et al.* 2014; Morrison *et al.* 2016). Rare studies on ants or ant–plant mutualism in West Africa focus on early natural history of the role of ants in cocoa agroforestry in Ghana and Nigeria (Strickland 1951; Leston 1970; Adenuga 1975; Majer 1976) with a few sampling in forest habitats (Belshaw and Bolton 1993, 1994). Recent work focussed on the role of weaver ant *Oecophylla longinoda* as biological control in orchards in Benin (Ouagoussounon *et al.* 2013; Vayssières *et al.* 2015; Wargui *et al.* 2015; Wetterer 2017) but our understanding of ant defence mutualism in this region is still limited.

According to their aggressiveness and efficiency in defending trees against elephants, African acacia ants were classified into dominant (*Crematogaster sjostedti*), intermediate (*C. mimosae* and *C. nigriceps*) and subordinate (*Tetraponera penzigi*) species (Palmer *et al.* 2013). The aggressiveness that underlies the defensive effects of ants against elephants also enables them to outcompete other ants (Heil 2013). The role played by ants in terrestrial ecosystems is always altered after human disturbance. In savannas, the species composition of ant communities varies across fire regimes (Parr *et al.* 2004; Ratchford *et al.* 2005; Vasconcelos *et al.* 2008, 2017; Maravalhas and Vasconcelos 2014). Fire destroys many ant populations and can reduce ant diversity and abundance in terrestrial ecosystems, and ultimately contribute to the loss of the rare ant species and or limit their aggressiveness. In some *Acacia*–ant systems, fire management can shift plant–ant occupancy. For example, many *A. drepanolobium* trees lost their ant colonies after burns (Kimuyu *et al.* 2014; Pringle *et al.* 2015). Fire-induced colony mortality was greatest for small trees (Kimuyu *et al.* 2014). Contrary to arboreal ants, fire increased the mean number of ant species on the ground within neotropical savannas (Frizzo *et al.* 2012). Without their ant colonies, individuals of the myrmecophyte *A. drepanolobium* were equally browsed as the typically non-myrmecophyte *Acacia mellifera* trees which experience catastrophic elephant herbivory (Goheen and Palmer 2010). Such tree vulnerability to elephant damages, due to increased fire, may have profound implications for population viability because *Acacia* adult trees have relatively low resprouting capacity (Morrison *et al.* 2016). Individuals of *A. mellifera* remain non-myrmecophyte even when surrounded by individuals of the myrmecophyte *A. drepanolobium* (Goheen and Palmer 2010) indicating that not all *Acacia* species engage in mutualistic defence.

Understanding the ecological interactions between ant communities and *Acacia* damage by elephants is important for the sustainable management of these savanna ecosystems. In West Africa, the Biosphere Reserve of Pendjari is an ideal area for investigating *Acacia*–ant mutualism against elephant herbivory because the geomorphology of the reserve does not create natural refuge for tree species to escape elephant damage (Kassa *et al.* 2014). Unlike East African protected areas, large extensions of the savannas in the Pendjari are annually burnt (Azihou *et al.* 2013) and dominated by species belonging to the Combretaceae family (Assédé *et al.* 2012). Early fires are used yearly by the management of the Biosphere reserve to promote grass resprout which attracts wildlife closer to roads for viewing tourism while limiting higher rate of topkill for the woody species. Eleven *Acacia* species occur in the reserve including *Acacia sieberiana*, *Acacia seyal*, *Acacia gourmaensis*, *Acacia hockii* and *Acacia dudgeonii* which have been reported to be highly browsed by elephants (Azihou 2008; Assédé *et al.* 2012). The Pendjari Biosphere Reserve supports 869 elephants with a density of 1 individual per 6 km^2^ (Bouche *et al.* 2011).

In this study, we assessed how the composition and diversity of ant species associated with *Acacia* trees in the Biosphere Reserve of Pendjari impact elephant damage. Because all management units of the reserve experience the same fire regime, we hypothesize that ant community will be similar across management units. We expect annual management fires to favour the richness of ground-dwelling ant species while hindering the establishment of arboreal ants on *Acacia* trees especially on smaller individuals. Following the competitive exclusion hypothesis, we expect a single ant species to be abundant on myrmecophyte *Acacia* individuals leading to rare co-occurrence of *Acacia* ants on the same tree. As observed in East African savannas, we finally predict individuals of non-myrmecophyte *Acacia* species to suffer higher attack rates from elephants than would ant-inhabiting *Acacia* species. Testing such hypotheses in the Biosphere Reserve of Pendjari will provide new evidence on the effect of fire on *Acacia*–ant mutualism while filling the knowledge gap between Eastern and Western Africa on the tritrophic interaction between elephants, ants and *Acacia* species.

## Materials and Methods

### Study site

The Pendjari Biosphere Reserve is the uppermost north-west Benin in the Sudanian region of the Dahomey Gap (Adomou *et al.* 2006; Assédé *et al.* 2012) between the latitudes 10°30′ and 11°30′N and the longitudes 00°50′ and 2°00′E. The reserve covers an area of 4661.4 km^2^ and includes the National Park of Pendjari or core zone (2660.4 km^2^) and the hunting zones (hunting zone of Pendjari: 1750 km^2^ and hunting zone of Konkombri: 251 km^2^). The core zone is a strictly protected area where the vegetation is less disturbed by anthropogenic activities; only ecotourism is allowed. The core zone and the hunting zones are surrounded by a buffer zone where human activities are under control (Assédé *et al.* 2012). These management units experience early vegetation fire on a yearly basis. Hunting elephants is prohibited in these management units. The climate of the Biosphere Reserve of Pendjari is characterized by one rainy season (April/May to October) and one dry season (November to March). The total annual rainfall averages 1000 mm with 60 % falling between July and September. During the rainy season, large parts of the reserve are flooded. The mean annual temperature is 27 °C. In addition, the relative humidity varies between 17 and 99 % over the year (Azihou 2013). The eastern part of the reserve is bordered by the Atacora mountain chain (400–513 m above sea level). Large isolated hills and floodplains are also present. The reserve is mainly characterized by tropical ferruginous soils except on hills (rock outcrops) and in flooded zones (clayey soil and silty soil) (Willaine and Volkoff 1967). The Pendjari is the only important river in the reserve that carries water throughout the year. It runs through the National Park of Pendjari and the Pendjari hunting zone. The vegetation of the Biosphere Reserve of Pendjari is dominated by savannas (tree, shrub and grass savannas) with islands of woodlands, dry forests and gallery forests along rivers (Azihou 2013).

### Sampling of the diversity and abundance of ants

We measured the diversity and abundance of ants once during the dry season after the annual vegetation fire in stands of *A. sieberiana*, *A. seyal*, *A. gourmaensis*, *A. hockii* and *A. dudgeonii* in the Pendjari Biosphere Reserve. Data on ants’ abundance were collected on *Acacia* trees sampled in 28 plots of 100 m × 100 m each. We sampled ants in eight plots for *A. sieberiana* sites and in five plots for each of the other *Acacia* species because *A. sieberiana* was more represented in the Pendjari Biosphere reserve. To evaluate the abundance and diversity of the ants, we used attractive traps consisting of 30 × 30 cm white ceramic tiles, each with a 4 cm spot of bait composed of honey mixed with canned tuna at its centre. These attractive traps designed to evaluate the abundance and diversity of ants were each placed under the *Acacia* trees and deployed for 30 min before the ants were counted and collected with a mouth aspirator. Ant counting was perfected in digital photographs of the samples (Dassou *et al.* 2015, 2016). Bait traps were placed under 10 randomly chosen *Acacia* trees in each plot. To reinforce the collection of the diversity and abundance of ants, after collecting all the ant taxa in the bait trap we captured with an aspirator all ants that move on the *Acacia* tree stem above the trap to a height of 2 m. These ant taxa collected on the tree stem were counted at the lab. In plots with less than 10 *Acacia* trees, all individuals were sampled. All samples were obtained in the morning between 8:00 a.m. and 12:00 p.m. Ants caught were preserved in 70 % ethanol solution. Ant species collected in the *Acacia* ecosystems were identified at the Entomological Museum of the International Institute for Tropical Agriculture, Benin Station.

### Measurements of the intensity of elephant damage

We measured the intensity of elephant damage on the same *Acacia* trees on which ant diversity and abundance data were collected. On each sampled tree we measured the diameter at breast height (dbh), diameter of crown, total and trunk heights. The intensity of elephant damages was scored following the classification of Riginos *et al.* (2015). Two types of elephant damages were observed in the field: barking and branch breaking. The intensity of branch breaking was measured on a 1–5 scale: 1 for light or no damage (branch tips exhibit slight browsing); 2 for mild damage (branches browsed and broken); 3 for medium damage (20–50 % of canopy was destroyed); 4 for substantial damage (50–99 % of canopy was destroyed); and 5 for catastrophic damage (tree completely pushed over or 100 % canopy destroyed). We used experienced field guides to distinguish tree damaged by elephants from damages due to abiotic factors such as wind or storm. Bark damage was scored on a seven-level scale, from the lowest to the highest intensity (Ihwagi *et al.* 2012; Kassa *et al.* 2014): score 0 for undamaged, 1 for tusked, 2 for 1–25 % debarked, 3 for 26–74 % debarked, 4 for 75 % debarked, 5 for 76–99 % debarked and 6 for ring debarked.

### Data analysis

All statistical analyses were performed in R (R Core Team 2019). A permutational multivariate analysis of variance using distance matrices (ADONIS) was performed on the data on ant communities associated with *Acacia* species using the package *vegan* (Oksanen *et al.* 2015). The source of variation introduced in the analysis included the intensity of elephant damages, *Acacia* species (*A. dudgeonii*, *A. gourmaensis*, *A. hockii*, *A. seyal* and *A. sieberiana*) and ant sampling location (tree versus ground). For each ant species, indicator value (IndVal) scores were calculated from samples grouped according to *Acacia* species and ant sampling location (see Dufrêne and Legendre 1997 for details on IndVal calculations). The IndVal computed using the package *labdsv* (Roberts 2015) estimates the association of each ant species to each group (*Acacia* species or ant sampling location). IndVal is scaled from 0 to 100 % with a value of 100 % indicating that the ant species was collected in every sample within a group and not in any other group.

Co-occurrence patterns of ant species under *Acacia* trees and on *Acacia* trees were assessed by computing the frequency of observations where each species pair is jointly recorded. The results were presented using a *corrgram*, an advanced graphical tool (Zuur *et al.* 2010; Azihou *et al.* 2013). A general linear model was used to test for difference in the intensity of elephant damages among *Acacia* species. The Student–Newman–Keulh (SNK) test was performed using the package *agricolae* (De Mendiburu 2014) for pairwise means comparison. We performed a χ^2^ test to assess the independence between *Acacia* species and ant occurrence on trees. For each *Acacia* species, we used a Student’s *t*-test to compare the height and the intensity of elephant damages between trees with ants and trees without ants. A beta regression was performed using the package *betareg* (Cribari-Neto and Zeileis 2010) to test the effect of ant abundance and species richness under *Acacia* trees and on *Acacia* trees on the intensity of elephant damage. There was a significant correlation between ant abundance and species richness for observations made on *Acacia* trees. Therefore, we ran the analysis separately for each response variable.

## Results

### Abundance and diversity of ant species associated with *Acacia* trees

At total 8772 ants including 11 ant species were associated with *Acacia* trees namely *Monomorium bicolor* (3584 individuals), *Pheidole megacephala* (3412 individuals), *Pheidole rugaticeps* (852 individuals), *Camponotus sericeus* (372 individuals), *Trichomyrmex oscaris* (230 individuals), *Camponotus carbo* (147 individuals), *Plagiolepis alluaudi* (102 individuals), *Crematogaster coelestis* (29 individuals), *Myrmicaria opaciventris* (21 individuals), *Brachyponera sennaarensis* (20 individuals) and *Camponotus maculatus* (3 individuals). Most of these ant species are generalist with widespread distribution. Abundance and diversity of ant species did not vary across the intensity of elephant damages (*F*_1_ = 1.346, *P* = 0.196) contrary to *Acacia* species (*F*_4_ = 3.612, *P* = 0.001) and location of ant sampling (*F*_1_ = 5.751, *P* = 0.001) (Table 1). The interaction between the intensity of damages and *Acacia* species was not significant (*F*_4_ = 1.003, *P* = 0.416), suggesting that the abundance and diversity of ant species associated with an *Acacia* species did not vary according to the intensity of elephant damages. Similarly, the interaction between the intensity of damages and location of ant sampling was not significant (*F*_1_ = 1.355, *P* = 0.182). Thus, the abundance and diversity of ant species associated recorded under or on *Acacia* trees did not vary according to the intensity of elephant damages. The interaction between *Acacia* species and location of ant sampling was not significant (*F*_2_ = 1.123, *P* = 0.318) indicating that the assemblage of ants recorded under or on trees did not vary across *Acacia* species.

**Table 1.** Outputs of the permutational multivariate analysis of variance using distance matrices (ADONIS) on ant communities associated with *Acacia* species in the Biosphere Reserve of Pendjari. SS = sums of squares, MS = mean square, DF = degree of freedom.

Source of variation	DF	SS	MS	*F*	*R* ^2^	*P*
Intensity of damages	1	0.481	0.481	1.346	0.006	0.196
*Acacia* species	4	5.168	1.292	3.612	0.063	0.001
Ant location	1	2.057	2.057	5.751	0.025	0.001
Intensity of damages × *Acacia* species	4	1.435	0.359	1.003	0.018	0.416
Intensity of damages × ant location	1	0.485	0.485	1.355	0.006	0.182
*Acacia* species × ant location	2	0.804	0.402	1.123	0.010	0.318
Intensity of damages × *Acacia* species × ant location	2	0.286	0.143	0.400	0.003	0.986
Residuals	199	71.189	0.358	0.869		
Total	214	81.904			1	

Five ant species were identified as indicator of *Acacia* species and location of ant sampling (Table 2). *Camponotus sericeus* (IndVal = 0.414, *P* = 0.028) and *C. coelestis* (IndVal = 0.088, *P* = 0.008) showed a substantial fidelity with the arboreal habitat. *Monomorium bicolor* (IndVal = 0.259, *P* = 0.003) and *P. rugaticeps* (IndVal = 0.200, *P* = 0.001) were most associated with *A. gourmaensis* trees while *C. carbo* (IndVal = 0.166, *P* = 0.008) showed a significant fidelity with *A. dudgeonii* individuals.

**Table 2.** Indicator species analysis on significant factors identified through ADONIS

Parameter	Ant species	Indicator value	*P*-value
Ant location = on tree	*Camponotus sericeus*	0.414	0.028
Ant location = on tree	*Crematogaster coelestis*	0.088	0.008
*Acacia* species = *Acacia gourmaensis*	*Monomorium bicolor*	0.259	0.003
*Acacia* species = *Acacia gourmaensis*	*Pheidole rugaticeps*	0.200	0.001
*Acacia* species = *Acacia dudgeonii*	*Camponotus carbo*	0.166	0.008

### Co-occurrence among ant species under *Acacia* trees and on host plants

**Figure 1.**
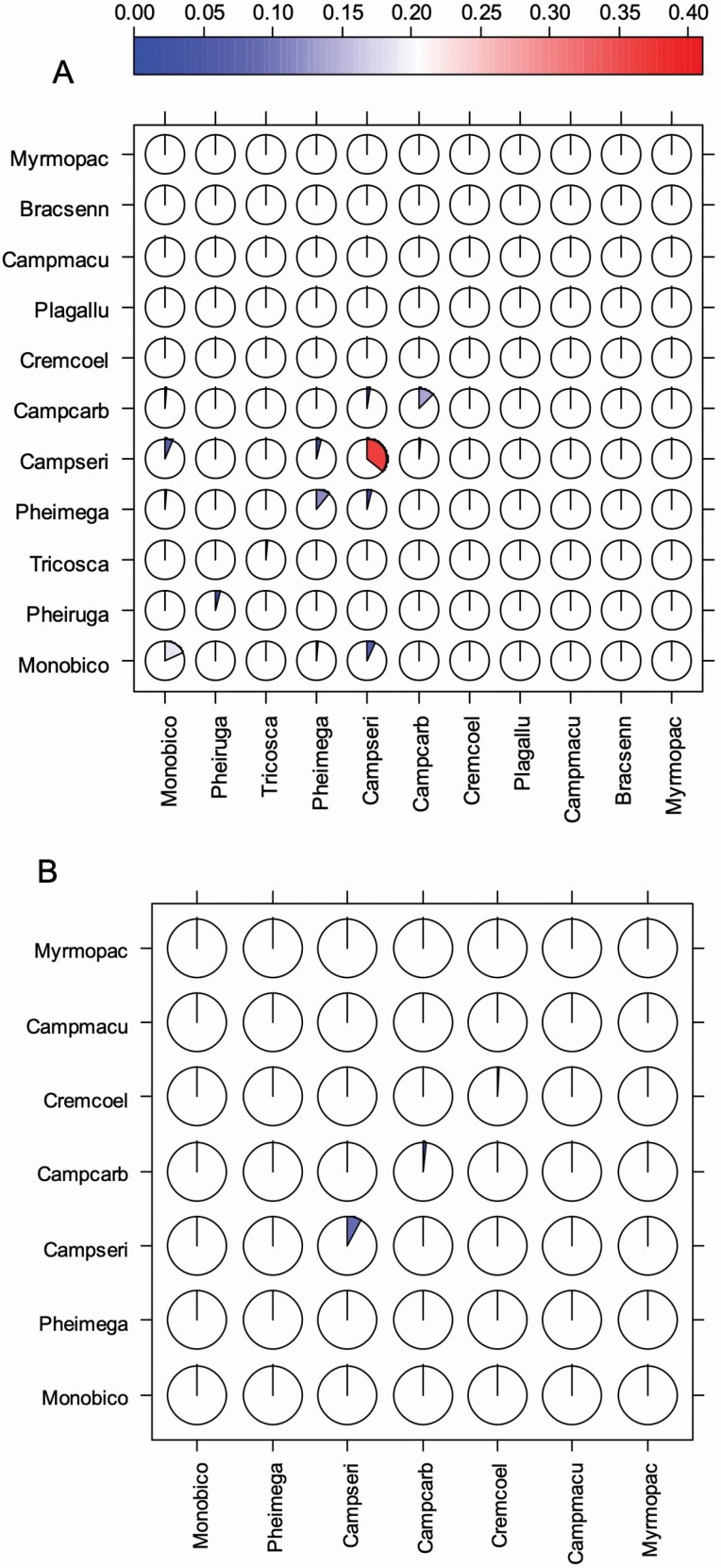
Corrgram showing the frequency with which pairs of ant species both occurred (A) under the same *Acacia* tree and (B) on the same *Acacia* tree. The colour and the amount that a circle has been filled correspond to the proportion of joint presence observations. The diagonal running from the bottom left to the top right represents the percentage of trees where each ant species was observed. Eight-letter acronyms represent the different tree species. Full species names: Monobico = *Monomorium bicolor*, Pheiruga = *Pheidole rugaticeps*, Tricosca = *Trichomyrmex oscaris*, Pheimega = *Pheidole megacephala*, Campseri = *Camponotus sericeus*, Campcarb = *Camponotus carbo*, Cremcoel = *Crematogaster coelestis*, Plagallu = *Plagiolepis alluaudi*, Campmacu = *Camponotus maculatus*, Bracsenn = *Brachyponera sennaarensis*, Myrmopac = *Myrmicaria opaciventris*. The legend bar relates the colours in the graph to the proportion of the observations.

All ant species were recorded under the 261 sampled *Acacia* trees (Fig. 1A). Except *C. sericeus* (36 %) and *M. bicolor* (19 %), *C. carbo* (13 %) and *P. megacephala* (12 %), ant species were successfully sampled under less than 5 % of *Acacia* trees. *Camponotus maculatus*, *B. sennaarensis* and *M. opaciventris* did not co-occur with any ant species. For the remaining species, the highest co-occurrence frequency equalled 7 % and was observed between *M. bicolor* and *C. sericeus* mainly under *A. dudgeonii* and *A. seyal* individuals. These species, respectively, co-occurred with six and five other ant species.

Only seven ant species were recorded on 12.64 % of the sampled *Acacia* trees (Fig. 1B) namely *M. bicolor*, *P. megacephala*, *C. sericeus*, *C. carbo*, *C. coelestis*, *C. maculatus* and *M. opaciventris*. These species occurred on less than 2 % of sampled *Acacia* trees except *C. sericeus* (9 %). *Monomorium bicolor*, *P. megacephala* and *M. opaciventris* were not jointly recorded on any *Acacia* trees with any other ant species. Co-occurrence of ant species pair was rare and included only *C. coelestis*–*C. carbo* (0.8 %), *C. coelestis*–*C. sericeus* (0.4 %), *C. carbo*–*C. sericeus* (0.4 %) and *C. carbo*–*C. maculatus* (0.4 %).

### Ant species and elephant damage on *Acacia* trees

Across *Acacia* tree species, branch breaking was the main elephant damage observed on 94–100 % of sampled individuals (Table 3). The intensity of elephant-driven branch breaking significantly varied among *Acacia* tree species (*F*_4,251_ = 2.849, *P* = 0.025). Individuals of *A. sieberiana* experienced the highest damage (19 % of branch broken) contrary to *A. dudgeonii* (10 % of branches broken). Ant species were absent on *A. dudgeonii* and *A. hockii* trees. Observation of ants on trees was 2-fold higher for *A. sieberiana* (29 % of sampled trees) than *A. gourmaensis* and *A. seyal* (14 %). However, this variation was not significant (χ^2^_2_ = 4.655, *P* = 0.098). *Acacia gourmaensis* trees without ants were significantly smaller than their congener with ants (mean total height equals to 6.36 and 7.62 m, respectively; *t*_44_ = −2.050, *P* = 0.046). Individuals of *A. seyal* showed a similar but not significant trend (5.72 and 6.65 m, respectively; *t*_45_ = −1.862, *P* = 0.069). The total height did not vary between *A. sieberiana* trees without and with ants (11.79 and 11.26 m, respectively; *t*_63_ = 0.767, *P* = 0.446). The mean total height within the two *Acacia* species (*A. dudgeonii* and *A. hockii*) whose trees have no ants was statistically equalled to that of *A. gourmaensis* individuals without ants (5.74, 6.23 and 6.36 m, respectively; *F*_2,97_ = 1.985, *P* = 0.143).

**Table 3.** Elephant damages on *Acacia* tree species within the Biosphere Reserve of Pendjari. Numbers followed by the same letter are not different at 5 % level.

*Acacia* species	*N*	Elephant damages (%)				Host plants (%)
		Barking		Branch breaking		
		Frequency	Intensity	Frequency	Intensity	
*Acacia sieberiana*	65	0	NA	100	18.98 ± 1.66a	29.23
*Acacia gourmaensis*	49	6.12	23.33 ± 3.33	93.88	14.98 ± 1.79ab	14.29
*Acacia hockii*	48	0	NA	100	14.77 ± 2.67ab	0.00
*Acacia seyal*	49	4.08	45.00 ± 5.00	95.92	13.60 ± 2.95ab	14.29
*Acacia dudgeonii*	50	0	NA	100	9.74 ± 0.83b	0.00

The abundance of each ant species per tree varied between 1 and 8.33 except one observation of 500 individuals of *M. bicolor* on an *A. gourmaensis* tree (Table 4). Overall, the intensity of elephant-caused branch breaking did not vary between trees with ants and trees without ants: *A. sieberiana* (20.68 and 18.28 %, respectively; *t*_63_ = −0.654, *P* = 0.516), *A. gourmaensis* (12.14 and 15.49 %, respectively; *t*_44_ = 0.666, *P* = 0.509), *A. seyal* (7.00 and 14.75 %, respectively; *t*_45_ = 0.933, *P* = 0.356). Neither the abundance of ants on *Acacia* trees, nor their species richness had a significant effect on the intensity of elephant-caused branch breaking across the five *Acacia* species (Table 5). A similar trend was observed for ants recorded under *Acacia* trees except *A. dudgeonii* where the abundance of ants under trees significantly reduced branch breaking by elephants (*Z* = −3.029, *P* = 0.002).

**Table 4.** Abundance (mean ± SE) of each ant species on *Acacia* trees within the Biosphere Reserve of Pendjari.

Ant species	*Acacia sieberiana*		*Acacia gourmaensis*		*Acacia seyal*	
	Frequency	Abundance (mean ± SE)	Frequency	Abundance (mean ± SE)	Frequency	Abundance (mean ± SE)
*Pheidole megacephala*	1	5.00	0	NA	0	NA
*Monomorium bicolor*	0	NA	1	500.00	0	NA
*Camponotus sericeus*	12	3.50 ± 0.925	6	4.67 ± 0.558	5	1.60 ± 0.245
*Camponotus carbo*	4	6.00 ± 3.674	0	NA	2	1.50 ± 0.500
*Myrmicaria opaciventri*	2	5.50 ± 0.500	0	NA	0	NA
*Crematogaster coelestis*	3	8.33 ± 3.333	0	NA	0	NA
*Camponotus maculatus*	1	1.00	0	NA	0	NA

**Table 5.** Estimates of the beta regression models on the variation of the intensity of elephant-caused branch breaking according to the abundance and species richness of ants on host trees and under *Acacia* trees.

Models	Variables	Tree species														
		*Acacia sieberiana*			*Acacia gourmaensis*			*Acacia hockii*			*Acacia seyal*			*Acacia dudgeonii*		
		Coef	*z*	*P*	Coef	*z*	*P*	Coef	*z*	*P*	Coef	*z*	*P*	Coef	*z*	*P*
Ants on trees																
Model 1	Abundance	−0.010	−0.365	0.715	−0.001	−0.817	0.414	NA			−0.167	−0.589	0.556	NA		
Model 2	Richness	−0.038	−0.214	0.830	−0.240	−0.768	0.442	NA			−0.312	−0.664	0.507	NA		
Ants under trees																
Model 3	Abundance	0.003	1.262	0.207	0.002	0.368	0.713	0.010	0.476	0.634	−0.019	−0.439	0.661	−0.013	−3.029	0.002
	Richness	−0.050	−0.441	0.659	−0.230	−1.161	0.246	−0.008	−0.035	0.972	−0.006	−0.022	0.983	0.066	0.450	0.653
	Abundance × richness	−0.002	−1.208	0.227	−0.002	−0.369	0.720	−0.009	−0.476	0.634	0.011	0.409	0.683	0.012	2.971	0.003

## Discussion

Within the annually burnt reserve, ants were more diverse and frequent beneath than on *Acacia* trees, consistent with the contrasting effects of fire on arboreal and ground-dwelling ant communities. Fire can reduce the number of ant species on trees and increases species richness on the ground (Frizzo *et al.* 2012). Moreover, there were few trees with ants and for some species (*A. gourmaensis* and *A. seyal*), empty trees were significantly smaller on average than trees with ants. This is in line with the greatest fire-induced ant colony mortality observed on *A. drepanolobium* (Kimuyu *et al.* 2014). The absence of ants on individuals of *A. dudgeonii* and *A. hockii*, which are the smallest *Acacia* species in our study area, may be a legacy of such fire-induced tree ant mortality in this annually burnt habitat. This effect was not observed for *A. sieberiana* which had the taller individuals, indicating that tree height can mediate the effect of fire on ant colonies in frequently burnt habitats. Studies on plant–ant mutualism are rare in West Africa (but see Adenuga 1975; Majer 1976; Belshaw and Bolton 1994) and our understanding of the limitation of ant–*Acacia* association in this part of the world is limited. Perhaps, the low ant residency on *Acacia* species in our study system, which is frequently burned, is related to potential disruptive effect of frequent fire in the Pendjari Biosphere Reserve. Fire may kill or weaken host trees and thereby indirectly limit ant colonization (Janzen 1967). Fire may also directly limit ant occupancy (Cochard and Agosti 2008). For example, in the Kenya Long-term Exclosure Experiment, survival of arboreal ant colonies was directly affected by fire with colonies on taller trees, which are out of reach of fire, surviving better (Kimuyu *et al.* 2014).

We found limited co-occurrence of ant species on the *Acacia* host trees we studied. The joint presence of two ant species was observed on only 2 % of the sampled *Acacia* trees. This is also common in East African savannas. For *A. drepanolobium*, there is a linear hierarchy in competition among four *Acacia* ant species for nesting space (Palmer *et al.* 2013): *C. sjostedti* > *C. mimosa* > *C. nigriceps* > *T. penzigi*. In this hierarchical structure, competition occurs via direct takeover of host plants by neighbouring colonies. Among the recorded ant species within the Biosphere Reserve of Pendjari, only *C. coelestis* belong to the same genus as three previous *Acacia* ants reported in East Africa (Palmer *et al.* 2013).

As expected, the intensity of elephant damage varies among *Acacia* species with *A. sieberiana* as the most damaged species. This is congruent with our predictions that individuals of non-myrmecophyte *Acacia* species suffer higher attack from elephants. With no record of ants on trees, *A. dudgeonii* was the less damaged species. This species may be less palatable. Similar trends were observed in Serengeti National Park where elephant highly damaged *A. senegal* compare to *Acacia robusta*, *Acacia tortilis*, *Acacia gerradii* and *A. drepanolobium* (Morrison *et al.* 2016). Ant assemblage, abundance or richness did not limit the intensity of elephant damage on trees in the Biosphere Reserve of Pendjari. The low density of ants observed on trees in our study region may explain this pattern. Indeed, high density of ants on host plants (up to 90 000 workers on some trees) is an important component of their defensive efficacy (Goheen and Palmer 2010). The ecology and behaviour of ant species caught on trees may also explain the observed trends. For instance, *C. sericeus*, the most frequent species observed on *Acacia* trees is a ground-nesting and a non-aggressive ant species that regularly visit extra-floral nectaries of savanna trees (Mody and Linsenmair 2003). Similarly, *C. maculatus* (Blard *et al.* 2003), *P. megacephala* (Seguni *et al.* 2011; Visitacao 2011) and *M. opaciventris* (Kenne and Dejean 1999) are ground-nesting ant species. Moreover, *P. megacephala* is an invasive ant (Wetterer 2012) that increases elephant damage to *Acacia* trees through the disruption of protective ant–plant mutualism (Riginos *et al.* 2015). Ant species falling to protect *Acacia* trees from elephant damage is not typical of the Biosphere Reserve of Pendjari. Even in East Africa where obligate *Acacia*–ant mutualism is prevalent (Palmer and Brody 2013), many ant species (e.g. *Crematogaster nigriceps* and *T. penzigi*) provide low protection against mega-herbivores (Martins 2010; Palmer *et al.* 2013). Some *Acacia* trees are inhabited by non-defending exploiter ants (Heil 2013). Myrmecophytes may be able to persist even in the absence of their mutualistic ant in habitats with limited herbivory and competition (Janzen 1973) or potentially in rich habitats where the cost of biomass reconstruction is limited (Coley *et al.* 1985; Endara and Coley 2011). However, even in the absence of such direct defence role, ants may indirectly influence myrmecophytes. Several ant species tend hemipterans which exert biological control influence on plant herbivores (Martins 2013), provide additional dry season food supplement that maintains ant colonies (Prior and Palmer 2018) or by developing beneficial association with fungi and microbes (Baker *et al.* 2017).

All in all, annual vegetation fire hinders the establishment of ants on small *Acacia* species (*A. gourmaensis*, *A. seyal*, *A. dudgeonii* and *A. hockii*). The taller species (*A. sieberiana*) that escape such firetrap falls to attract ant colonies while experiencing high elephant damages. This species may be qualified as non-myrmecophyte. *Acacia* species that do not engage in ant mutualism respond to browsing by large African herbivores with physical and chemical defences. In Game Ranching Ltd (central Kenya), individual *A. seyal* exposed to intensive browser utilization was observed to lose shoot tips, produce long thorns and have relatively few flowers and fruits (Milewski and Madden 2006). Similarly, *A. sieberiana* trees with high browsing intensity had significantly longer spines, smaller leaves and higher total cyanide (prussic acid) concentrations than trees with low browsing intensity (Zinn *et al.* 2007). Further investigations are required to quantify such defence mechanisms and their effectiveness to protect *Acacia* trees against elephant damage in the Biosphere Reserve of Pendjari.

## Data Availability

The data and scripts used for the analyses in this paper are published online on Dryad Digital Repository at https://doi.org/10.5061/dryad.00000003t (Gaoue *et al.* 2021).

## Notes

**Populations & Communities. Chief Editor:** Jean Burns
